# Translating macroecological models to predict microbial establishment probability in an agricultural inoculant introduction

**DOI:** 10.3389/frmbi.2024.1452476

**Published:** 2024-10-02

**Authors:** Isaac M. Klimasmith, Bing Wang, Sora Yu, Yasuo Yoshikuni, Angela D. Kent

**Affiliations:** ^1^ University of Illinois Urbana-Champaign, Department of Crop Sciences, Urbana, IL, United States; ^2^ Department of Energy (DOE), Center for Advanced Bioenergy & Bioproducts Innovation, University of Illinois at Urbana-Champaign, Urbana, IL, United States; ^3^ Center for Quantitative Life Sciences, Oregon State University, Corvallis, OR, United States; ^4^ US Department of Energy Joint Genome Institute, Lawrence Berkeley National Laboratory, Berkeley, CA, United States; ^5^ Department of Natural Resources and Environmental Science, University of Illinois Urbana-Champaign, Urbana, IL, United States

**Keywords:** microbial inoculants, invasion ecology, propagule pressure, agriculture, modeling

## Abstract

The use of potentially beneficial microorganisms in agriculture (microbial inoculants) has rapidly accelerated in recent years. For microbial inoculants to be effective as agricultural tools, these organisms must be able to survive and persist in novel environments while not destabilizing the resident community or spilling over into adjacent natural ecosystems. Despite the importance of propagule pressure to species introductions, few tools exist in microbial ecology to predict the outcomes of agricultural microbial introductions. Here, we adapt a macroecological propagule pressure model to a microbial scale and present an experimental approach for testing the role of propagule pressure in microbial inoculant introductions. We experimentally determined the risk-release relationship for an IAA-expressing *Pseudomonas simiae* inoculant in a model monocot system. We then used this relationship to simulate establishment outcomes under a range of application frequencies (propagule number) and inoculant concentrations (propagule size). Our simulations show that repeated inoculant applications may increase establishment, even when increased inoculant concentration does not alter establishment probabilities. Applying ecological modeling approaches like those presented here to microbial inoculants may aid their sustainable use and provide a monitoring tool for microbial inoculants.

## Introduction

The use of potentially beneficial microorganisms in agriculture (microbial inoculants) has rapidly accelerated in recent years (Waltz, 2017), constituting widespread species introductions. Microbial inoculants are applied to enhance many aspects of crop production, including increasing nutrient availability to plants, maximizing nitrogen fixation by the rhizosphere community, increasing plant tolerance to stress, and improving pathogen resistance (Calvo et al., 2014). For microbial inoculants to accomplish these aims, they must be able to persist in the new environments they are introduced into. A successful microbial inoculant must be able to outcompete, antagonize, or occupy a novel niche to survive introduction and establish itself in the diverse soil community (Mawarda et al., 2020). The inoculant must also be able to rapidly proliferate from small numbers in an environment that may not be its native habitat. Essentially, a persistent inoculant is a good invader (Bell and Tylianakis, 2016).

Given the conceptual similarity between an invasive species and microbial inoculation, we aimed to adapt a macroecological invasion ecology model to a microbial context and demonstrate how this modeling technique could be used to monitor and predict the outcomes of microbial introductions. These techniques are needed because unpredictable inoculant establishment poses a barrier to successfully deploying microbial inoculants as agricultural inputs (Kaminsky et al., 2019; Albright et al., 2022; O’Callaghan et al., 2022). Albright and colleagues present a framework for inoculant introductions grounded in invasion ecology, in which three factors determine inoculant success: propagule pressure, environmental filtering, and biotic interactions (Catford et al., 2009; Albright et al., 2022). Of these factors, propagule pressure is entirely under human control.

Propagule pressure collectively encapsulates the number of introduced organisms (propagule size, PS), the number of introductions (propagule number, PN), and the probability of establishment per released individual (the risk/release relationship) (Lockwood et al., 2005; Cassey et al., 2018). In the context of agricultural microbial inoculants, the propagule size and propagule number are controlled, while the risk/release relationship is often unknown. In a single growing season, propagule size would correspond to the concentration of the inoculant (often reported in CFU), and the propagule number would correspond to the number of applications per season.

Recent publications in conservation ecology may offer tools to understand the impact of microbial inoculant application methods on the establishment of these inoculants. A model was developed in 2021 by Stringham and Lockwood to predict the establishment probability of an invasive species from propagule pressure data (Stringham and Lockwood, 2021). While this model was built to predict the invasion dynamics of plants, invertebrates, and vertebrates, propagule pressure also plays a role in microbial invasions (Acosta et al., 2015).

Both in-silico models and laboratory experiments have been used to investigate the dynamics of microbial invasions. These experiments have considered the number of introduced organisms, resident community composition and diversity (Roy et al., 2013; Jones et al., 2017; Rivett et al., 2018; Vila et al., 2019), ecological niches in the resident and introduced communities (Mallon et al., 2018; Li et al., 2019), evolution within invader and host communities (Vila et al., 2019), resource availability and supply (Mallon et al., 2015; Yang et al., 2017), and chemically-mediated interactions between invaders and residents (Kurkjian et al., 2021). However, while many of these experiments have studied the impact of varying *numbers* of introduced bacteria on invasion success (propagule size), few have examined the second component of propagule pressure: the number of introductions (Wang et al., 2021; Papin et al., 2024). In an agricultural system, this facet of propagule pressure is essential as commercial inoculants are applied at least once annually (Jambhulkar et al., 2016), resulting in repeated biological introductions in highly distributed and resource-rich agroecosystems.

**Figure 1:**
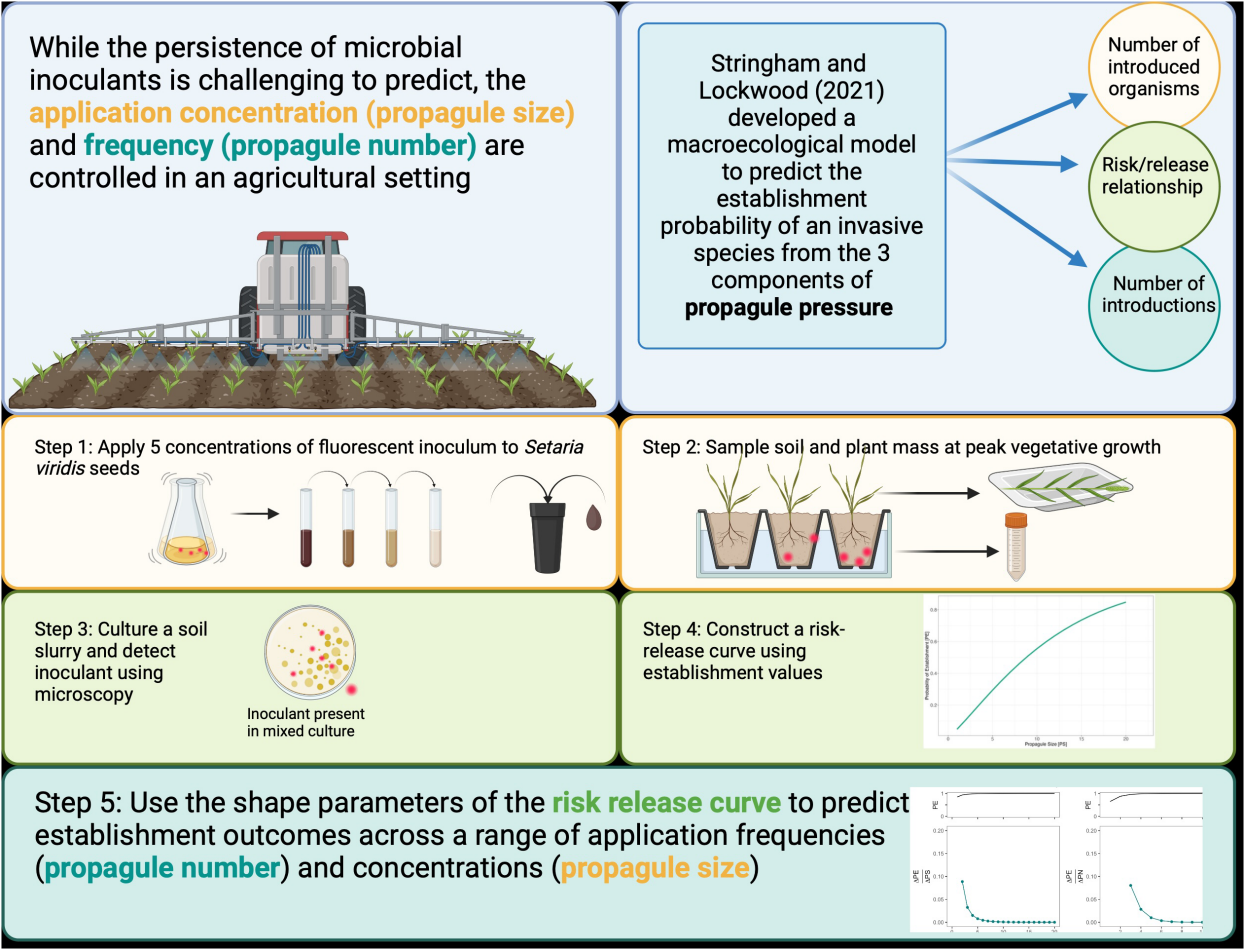
Graphical summary of background and methods. The three components of propagule pressure are colored-coded throughout.

To incorporate both the application density of an inoculant (propagule size) and the number of introductions (propagule number) into a predictive tool for agricultural inoculant introductions, we applied Stringham and Lockwood’s model to the establishment dynamics of *Pseudomonas simiae* in a model monocot (*Setaria viridis*) system (**Figure 1**). *S. viridis* is a close relative of grasses such as sorghum, switchgrass, and Miscanthus (Petti et al., 2013). As interest in these crops as bioenergy feedstocks increases, there is a growing focus on using microbial inoculants in their production (Fei et al., 2019). While the inoculant used here is generally regarded as a plant-growth-promoting bacteria (PGBR) (Desrut et al., 2020), we used a strain that had been genetically modified to express indoleacetic acid (IAA). Indoleacetic acid is a plant hormone widely studied in plant inoculation experiments for its effect on plant growth regulation and stress response. However, by encouraging greater root exudation, IAA also influences the plant-microbe relationship (Duca and Glick, 2020).

Here, we established the risk-release relationship for modified *Pseudomonas simiae* and monitored inoculant persistence as propagule size (PS) varied. Then, we modeled how persistence values would change under a range of possible application frequencies (PN). Concurrently, we monitored plant biomass, height, and leaf number during vegetative growth to assess whether inoculant persistence correlated with plant growth traits.

## Methods

### Experimental design

Five treatments (stock inoculant concentration [S], inoculant dilution 1 [D1], inoculant dilution 2 [D2], inoculant dilution 3 [D3], and a heat-killed negative control [Neg]) were applied at planting to pots planted with *Setaria viridis*. We planted 30 pots at each treatment level to account for potential variability in germination rates, initially arranged in racks by treatments. After germination, the pots were rearranged into a Latin rectangle, with 20 pots per treatment level. Of these pots, 15 replicates were included in the final analysis due to plant death.

### Inoculant strain and cultivation

Our experiment used the strain *Pseudomonas simiae* WCS417r as our inoculant. Studying the establishment dynamics of bacterial inoculants is challenging, given that the inoculant must be somehow distinguished from the resident community in culture-based approaches or the capacity of real-time measurements is limited, given delays inherent to DNA sequencing (Romano et al., 2020). To circumvent these limitations, we selected a strain of *P. simiae* that was both characterized as a plant-growth-promoting bacteria (Desrut et al., 2020) and was further genetically modified to constitutively express indoleacetic acid (IAA) and the fluorescence marker mCherry, making visually tracking the strain in mixed culture plates possible. These strains were modified using chassis-independent recombinase-assisted genome engineering (CRAGE), allowing the new genes to be directly integrated into the bacterial chromosome (Wang et al., 2020). Each integration site included antibiotic selection markers for kanamycin and apramycin.

The strains were stored on Luria-Bertani (LB) plates with kanamycin and apramycin (LB + KM/AP) added as selective agents and stored at 4°C until three weeks before the experiment began. At this point, we selected a single colony from the storage plates and streaked for isolation in triplicate onto LB + KM/AP plates to create our working cultures. From these plates, we transferred colonies to liquid LB media and incubated these cultures with shaking at 28°C for 10 hours.

To prepare the inoculants for the greenhouse experiments, we removed 10ml of culture, centrifuged it, and washed the pellet with sterile tap water two times to a final volume of 5ml (thus concentrating the culture twofold). The negative control was prepared by autoclaving a separate set of cultures and washing them with the same method to remove residual LB salts. The number of colony-forming units per ml was determined using plate counts on LB + KM/AP plates from a 10-fold dilution series.

The three treatment concentrations were prepared through a 50-fold dilution series from the stock concentration for a total of 5 treatments: stock (1.28 × 10^7^ CFU/ml), dilution 1 (2.56 × 10^5^ CFU/ml), dilution 2 (5.12 × 10^3^ CFU/ml), dilution 3 (1.024 × 10^2^ CFU/ml) and a heat-killed negative control (0 CFU/ml). Each pot received one *S. viridis* seed and 100 *μl* of the designated inoculant treatment at planting.

### Greenhouse procedures and plant care

We used a mixture of 10% unfertilized live field soil and 90% steam-sterilized sandy loam in autoclaved sterilized “conetainers” (with a volume of 84 cm^3^ each) as our plant substrate. Two days before planting, all pots were filled with soil and watered to encourage the growth of the resident microbial community.

We used *Setaria viridis*, a close relative of bioenergy feedstock grasses like sorghum and miscanthus, as our model monocot (Brutnell et al., 2010). *S. viridis* (accession PI 687376) seeds were obtained from the USDA-ARS Germplasm Resources Information Network (GRIN). To mimic field practices, we did not sterilize the seeds before planting. After planting, all pots were gently misted with water. Subsequently, the pots and plants were watered every 1-2 days and fertilized twice a week with 20-20-20 fertilizer at a concentration of 100 ppm (Jiang et al., 2013). Greenhouse temperatures were set to 28°C during the day and 22°C at night, with lighting on for 12 hours daily when outside light intensity fell below 300 w/m^2^ and shading provided when outside light intensity was over 700 w/m^2^.

### Sampling

At peak vegetative growth (Junqueira et al., 2019), all pots were destructively sampled for inoculant persistence and plant traits. We collected ~20 g of soil from each pot for plate counts (stored at 4°C). We measured the height of each plant, recorded the number of leaves, and removed the above-ground biomass. The biomass was oven-dried in paper bags and subsequently weighed.

### Culturing and microscopy

We conducted plate counts from soil slurries to estimate colonization density and confirm persistence (Olsen and Bakken, 1987). Each sample was plated in triplicate by vortexing 1g of soil with 10 ml of sterile water. 100 *μ*l of this slurry was transferred to LB + KM/AP plates and spread using sterile glass beads. The plates were grown at room temperature (~25°C) for 72 hours before being sealed with parafilm and stored at 4°C before fluorescence imaging.

The plates were imaged on an AxioZoom V16 microscope (Zeiss) with an Alexa Fluor 568 filter. Fluorescent colonies were counted by hand. In keeping with Stringham and Lockwood’s binary model of persistence (Stringham and Lockwood, 2021), inoculant persistence was recorded if any fluorescent colonies were present (as a binary presence/absence state).

### Modeling methods

In probabilistic models, propagule pressure is defined as the number of introduction events (propagule number, PN), the number of individuals or colony-forming units (CFUs) per introduction (propagule size, PS), the probability of establishment per individual or CFU (PE), and the risk release relationship. The risk-release relationship is similar to a dose-response curve and is the relationship between propagule size and establishment probability for a given species (*w*). Stringham and Lockwood construct a probabilistic model for propagule pressure based on the risk-release relationship, which they define as a continuous probability distribution (a Weibull function) (Leung et al., 2004; Stringham and Lockwood, 2021). The risk-release relationship is given as:


(1) $$\text{w}=1-{\text{q}}^{{\text{PS}}^{\text{c}}}$$

Here, *w* is the establishment probability for a given introduction, *q* is the probability that an individual (here, a single CFU) will fail to establish, PS is the propagule size, and *c* is a shape parameter that best defines the establishment curve. Leung et al. use *c* to account for Allee effects in small populations to reflect reduced per capita growth in small populations (Leung et al., 2004).

First, we simulated the establishment values at each inoculation level using a random binomial distribution. Each inoculation level (1.28 × 10^7^, 2.56 × 10^5^, 5.12 × 10^3^, 1.024 × 10^2^, 0 CFU/ml) was natural log-transformed and assigned a probability of success when drawing from the distribution (0, 0.2, 0.4, 0.6 and 0.8, respectively). The resulting successes were then summed and divided by the number of replicates per treatment level (15) to generate the experimental probabilities of establishment.

**Figure 2:**
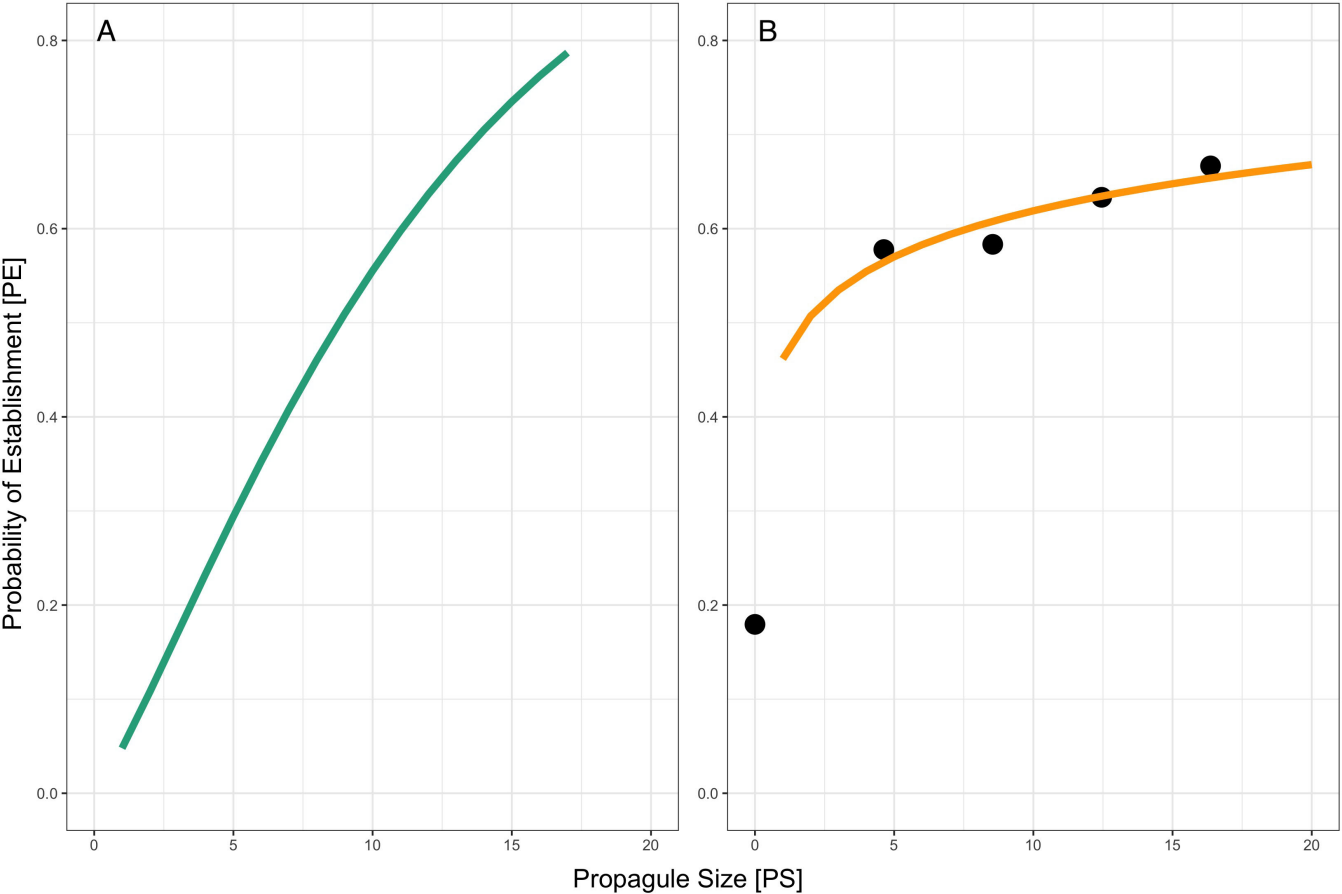
**(A)** Risk/release relationship of simulated establishment values, predicted using non-linear least squares and modeled using log-transformed experimental PS values (Equation 1). **(B)** Risk-release relationship for an experimental introduction (orange line) overlaid on observed persistence ratios (Equation 1). Propagule size (PS) corresponds to the natural log-transformed CFU/ml concentrations for each inoculation level. Experimental probability of establishment (black dots) is the proportion of pots within each treatment where the inoculant was detected.

Using the simulated probabilities of establishment at each level of inoculant concentration, we then used non-linear least squares to determine the shape parameters (**Figure 2A**). We used these parameters to fit a curve for the risk-release relationship, as defined in Equation 1. Next, we repeated this analysis using our experimental establishment ratios, producing an experimental risk-release curve (**Figure 2B**).

The shape parameters of this risk-release curve can then be applied to predict the outcome that changes to both propagule size and propagule number will have on establishment probability. The goal of this analysis in the context of conservation ecology would be to determine the maximum allowable numbers of introduced organisms (PS) and introductions (PN) that will keep the system below a target establishment probability to reduce the risk of invasion (Stringham and Lockwood, 2021). The target establishment has not been experimentally determined for commercial agricultural inoculants, so it was set between 0.5 and 0.8 in these simulations.

We applied the sensitivity analysis developed by Stringham and Lockwood to determine the impact of changing propagule size and number on establishment probability. Here, following Stringham and Lockwood’s method, we solve for the discrete partial derivatives of PS, PN, and q in Equation 1 using the backward difference method:


(2) $$\frac{\text{ΔPE}}{\text{ΔPS}}={\left({\text{q}}^{{\left(\text{PS}-1\right)}^{\text{c}}}\right)}^{\text{PN}}-{\left({\text{q}}^{{\text{PS}}^{\text{c}}}\right)}^{\text{PN}}$$

(3) $$\frac{\text{ΔPE}}{\text{ΔPS}}={\left({\text{q}}^{{\left(\text{PS}\right)}^{\text{c}}}\right)}^{\text{PN}-1}-{\left({\text{q}}^{{\text{PS}}^{\text{c}}}\right)}^{\text{PN}}$$

**Figure 3:**
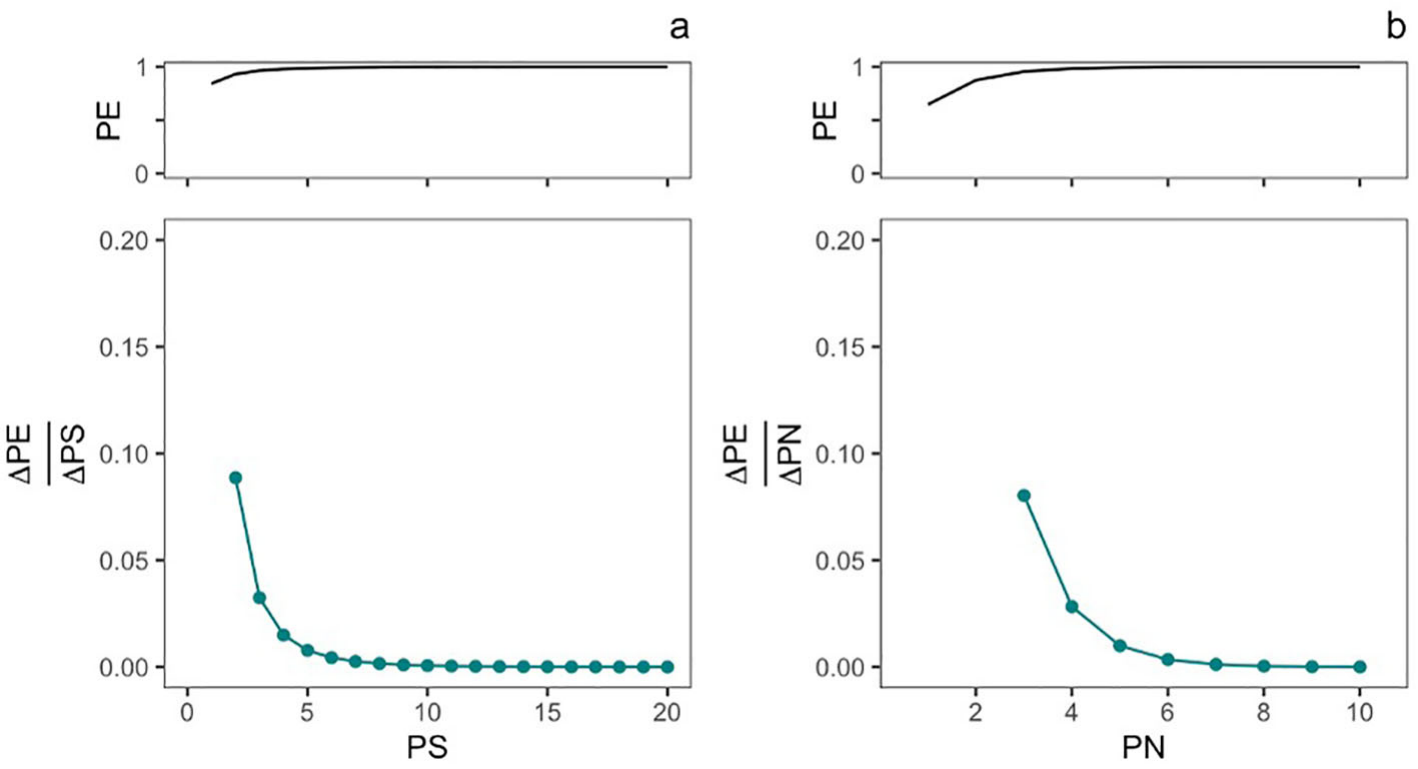
Predicted contribution of changes in propagule size (PS) and propagule number (PN) to inoculant persistence. The top panel of plot **(A)** shows the relationship between varying the propagule size from 1 (2.7182 CFU/ml) to 20 (4.85 × 10^8^ CFU/ml) on the probability of establishment (PE), while the number of introductions (PN) is held constant at 3. The bottom panel shows the sensitivity of PE to changes in PS. The top panel of plot **(B)** shows the relationship between varying propagule number (number of introductions, PN) from 1 to 10 introductions, while propagule size (PS) is held constant at 3.2690 × 10^6^ CFU/ml (log-transformed to 15). The bottom panel shows the sensitivity of PE to changes in PN.

We then implemented these equations using a modified version of the R simulation tool made available on GitHub by Stringham et al. and modeled two management options for a microbial introduction (**Figure 3**). In the first, the propagule number (PN) was held constant at three introductions, and we simulated an increase in propagule size from 2.7183 CFU/ml (log-transformed to 1) to 4.85 × 10^8^ CFU/ml (log-transformed to 20). In the second, propagule size (PS) was held constant at 3.2690 × 10^6^ CFU/ml (log-transformed to 15), and we simulated an increase in propagule number from 1 to 10 introductions. The original shape parameter *c* from the risk-release relationship was held constant in both simulations.

### Statistical methods

We used a robust heteroscedastic one-way ANOVA on trimmed means to test if mean plant height and biomass differed between inoculation treatments. We conducted *post hoc* pairwise comparisons with a Benjamini-Hochberg correction using the lincon() function in the WRS2 package (Mair and Wilcox, 2020). To test how leaf number depended on inoculation treatment, we used a log-linear (Poisson) regression. All statistical analyses were conducted in R, and all figures were produced using ggplot2 v3.4.1 (Wickham, 2016).

## Results

### Plant traits

**Figure 4:**
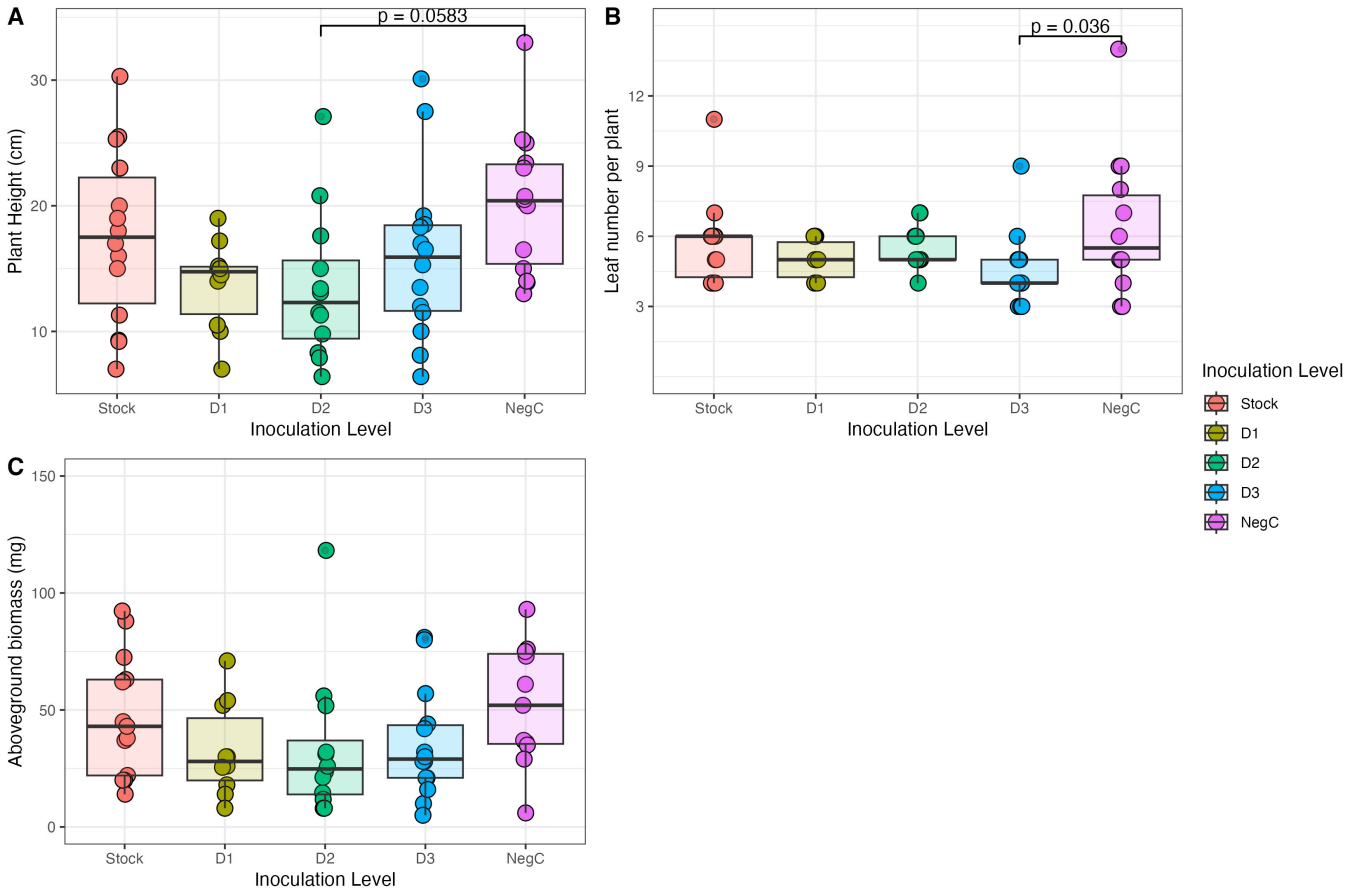
Plant height **(A)**, leaf number per plant **(B)** and aboveground biomass **(C)** at peak vegetive growth of *Setaria virdis* treated with 5 levels of inoculant treatment (Stock 1.28 × 10^7^ CFU/ml, D1–2.56 × 10^5^ CFU/ml, D2– 5.12 × 10^3^ CFU/ml, D3– 1.024 × 10^2^ CFU/ml and the negative control–0 CFU/ml).

Overall, inoculation did not significantly alter the plant traits measured here. Plant height ranged from 6.4 cm to 33.0 cm, with an overall median height of 15.25 cm. Across treatments, there was a significant reduction in plant height between plants treated with the negative control and D3 (**Supplementary Table 1**; **Figure 4**). Still, significant differences were not observed between other treatment pairs (as tested with *post-hoc* pairwise comparisons on a trimmed means ANOVA). Similarly, among the treatments, only D3 exhibited a statistically significant response (p = 0.0359), with fewer leaves than the negative control (**Supplementary Table 2**). Across all treatments, the median dried aboveground biomass was 35.5mg. Using a heteroscedastic one-way ANOVA, we fail to reject the null hypothesis that mean biomass differs between inoculant treatments (df = 4, F = 0.9397, p = 0.4632).

### Propagule pressure sensitivity analysis

Using the shape parameters of the experimental risk-release curve (*q*= 0.5384, *c*= 0.1927) (Stringham and Lockwood, 2021), we conducted a sensitivity analysis of the impact of propagule size and number on the establishment success of engineered *P. simiae* in a model monocot system (**Figure 3**). This analysis showed that PE was sensitive to changes in PN (number of introductions) for up until five introductions, after which PE became asymptotic. The modeled impact that changes in propagule size (number of released organisms) was only sensitivity to changes in PS at very low inoculant concentrations. Here, PE was sensitive to changes in propagule size until an inoculant concentration of about 148 CFU/ml (5 on the log-transformed scale).

## Discussion

As the efficacy of microbial inoculation depends on a degree of persistence, a key challenge in inoculant development is balancing persistence and potential for invasion (Kaminsky et al., 2019). This challenge is further complicated by the current lack of tools to monitor the status of microbial introductions or predict their outcomes (Mawarda et al., 2020; Jack et al., 2021). While a range of factors influence the fate of microbial introductions in agroecosystems, propagule pressure is directly controlled in the application process. To develop a method for predicting the outcome of agricultural microbial introduction, we applied a macroecological probabilistic propagule pressure model to an inoculated monocot system.

Based on a single introduction, we found that propagule size does not predict *P. simiae* establshment in an *S. viridis* system. By extrapolating from the risk-release curve, our models suggest that up to four repeated inoculations (increased propagule number) could increase microbial establishment success. The models include a term (the shape parameter *c*, see Equation 1) to model the Allee effect, the transition between a low growth rate at a low population size to a high growth once the population reaches a critical density (Leung et al., 2004; Taylor and Hastings, 2005; Stringham and Lockwood, 2021). While Allee effects have been observed in microbial populations (Kaul et al., 2016), the role of the Allee effect in purposeful microbial introduction has yet to be considered. In our models, the risk-release curve for *P. simiae* (modeled using the Allee effect) closely fits the observed persistence ratios (**Figure 2B**), indicating that including the Allee effect model aids in predicting the outcome of *P. simiae* introductions. By including this effect, the model can account for extremely dilute inoculants (low propagule size) or very low initial densities, which is useful given the low concentrations relative to soil volume used in field applications (Nerek and Sokołowska, 2022). However, including the Allee effect is less informative when comparing the establishment probability of highly concentrated or densely applied inoculants like those commonly used in greenhouse inoculation studies (Papin et al., 2024).

Although our models predict that multiple introductions may increase establishment success, these models assume that the risk-release curve exhibits the same shape parameters across multiple introductions. While the exact relationship between the number of introductions and persistence remains unclear, even transient introductions can alter the niche structure of the resident community (Mallon et al., 2018). If this is the case for microbial inoculant introductions, then each successive introduction could have a different *q* (probability that an individual will fail to establish) due to changes in the structure of the resident community, altering the parameters of the risk-release curve. Similarly, larger numbers of introduced bacteria could alter the competition dynamics with the resident community (Vila et al., 2019; Albright et al., 2020), altering the *q* and *c* parameters as propagule size increases. By keeping *q* and *c* static, these probabilistic models do not simulate changes in competition and resource use dynamics that have been observed in other experimental microbial invasions (Roy et al., 2013; Jones et al., 2017; Mallon et al., 2018).

Our modeling approach is simplified, using static establishment probabilities across introductions and a binary model for inoculant persistence. Currently, building a more complex model is challenging, given how little is known about the impact of multiple introductions on microbial establishment dynamics or how different application patterns interact with plant performance. In one of the few experimental forays into investigating both propagule size and propagule number, a recent study using *Pseudomonas fluorescens* demonstrated that while increased application frequency significantly improved inoculant survival at the 10-week mark, this difference was lost by the end of the experiment at 14 weeks (Papin et al., 2024). These results suggest that establishment probability may indeed fluctuate across multiple introductions, but likely does not follow a linear model. Furthermore, an ideal model for microbial propagule pressure would also include resident community composition, which was not investigated in our study.

The relative importance of microbial propagule pressure vs. community interactions is currently disputed. Some studies point to propagule pressure as the most important contributor to microbial invasion outcomes (Acosta et al., 2015), with resident community diversity playing a minor role (Kinnunen et al., 2018). Other studies contest these findings, showing that propagule pressure is a poor predictor of invasion outcomes (Albright et al., 2020). Generally, however, propagule size appears to interact with community composition, nutrient availability, and networks of competitive interactions to dynamically contribute to microbial invasion outcomes. While resident communities with high metabolic and phylogenic diversity are generally more resistant to invasion, very large propagule sizes can overcome this resident community resistance (Jones et al., 2017; King and Howeth, 2019; Li et al., 2019; Vila et al., 2019; Kurkjian et al., 2021). Environmental factors, such as nutrient availability and stress, further modulate this relationship, with invasion success increasing in high-nutrient, high-stress conditions (Roy et al., 2013; Li et al., 2022). Our findings demonstrate that propagule size and number contribute differently to invasion outcomes, with propagule size having little relative impact while propagule number may. From a management perspective, this could translate to tailoring microbial inoculant application methods based on the resident community’s susceptibility to invasion and the specific risk-release relationship for the inoculant.

In an agricultural setting, achieving repeated introductions would require a shift in inoculant application practices. Currently, inoculants are generally applied in a single application, such as with a liquid application (generally foliar or in-furrow at planting), on a seed coating, or alginate beads (Chaudhary et al., 2020). Achieving repeated introductions is challenging in many agricultural systems, particularly those that are not routinely irrigated or for which the ideal inoculant is not tolerant of a liquid suspension (Jambhulkar et al., 2016; Sahu et al., 2019). However, for those inoculants where a liquid suspension is viable, drip irrigation and repeated foliar sprays may offer a mechanism for repeated introductions (Boari et al., 2008).

Despite the high establishment success across a range of propagule sizes, successful inoculation with modified IAA-expressing *P. simiae* did not alter *S. viridis* biomass, leaf number, or height. Unmodified *P. simiae* is widely applied as a microbial inoculant and can help suppress soil pathogens, improve stress tolerance, increase root growth, and alter plant sugar transport pathways (Desrut et al., 2020; Pieterse et al., 2021). However, these responses can vary by plant genotype and may vary by species (Wintermans et al., 2016). Our plants were grown under ideal conditions, but *P. simiae* is especially beneficial in the presence of pests, pathogens, and environmental stressors (Pangesti et al., 2017; Chiappero et al., 2019; Loo et al., 2020; Pieterse et al., 2021). For *S. viridis*, the differential response between treated and untreated plants may emerge under stress, which was not tested here. The addition of IAA-producing microbial inoculants has been shown to increase root growth and nutrient uptake, improve drought and salt tolerance, and increase plant shoot length (Singh et al., 2013; Myo et al., 2019; Zarea, 2019; Peng et al., 2023). Independently of supporting plant growth, IAA production may improve bacterial survival and fitness (Kim et al., 2011), which could have aided in inoculant survival in our study. As both high microbial expression of IAA and *P.simiae* addition can modulate plant responses to stress, future work in this system should include studies of *S. viridis* performance under environmental stressors to determine the conditions under which inoculation is beneficial.

As the agricultural use of microbial inoculants continues to accelerate (Waltz, 2017), understanding the invasion ecology of these introduced species will be crucial to their sustainable use (Bell and Tylianakis, 2016; Jack et al., 2021). The persistence of microbial inoculants is currently highly variable and not consistently measured (Mawarda et al., 2020), making it challenging to predict the outcome of microbial introductions. We adapted a macroecological invasion ecology model to an agricultural microbial introduction and found that while propagule size may not predict establishment, propagule number may. While many previous studies have equated propagule size with propagule pressure, our findings emphasize the importance of evaluating all components of propagule pressure in microbial studies. As we move towards agricultural practices that incorporate microbial inputs, propagule pressure models can guide agricultural management decisions to help achieve target establishment probabilities that balance persistence and invasion.

## Data Availability

The datasets presented in this study can be found in online repositories. The names of the repository/repositories and accession number(s) can be found below: Illinois Data Bank, https://doi.org/10.13012/B2IDB-0907683_V1.
