# Supplementary material for: Translating macroecological models to predict microbial establishment probability in an agricultural inoculant introduction
Source: Front Microbiomes. 2024 Oct 2;3:1452476. doi: 10.3389/frmbi.2024.1452476 (PMC12993525; doi:10.3389/frmbi.2024.1452476)
Supplement: Supplementary file 1 [file Table1.docx]

| Contrast | Estimated difference | Lower CI (95%) | Upper CI (95%) | P value |
| --- | --- | --- | --- | --- |
| NegC vs. D1 | 5.81167 | -0.7545 | 12.37783 | 0.10783 |
| NegC vs. D2 | 7.345 | -0.02572 | 14.71572 | 0.05829* |
| NegC vs. D3 | 4.662 | -2.17052 | 11.49452 | 0.36281 |
| NegC vs. Stock | 2.455 | -6.49219 | 11.40219 | 0.54247 |
| D1 vs. D2 | 1.53333 | -5.20728 | 8.27395 | 0.54247 |
| D1 vs. D3 | -1.14967 | -7.16213 | 4.86279 | 0.54247 |
| D1 vs. Stock | -3.35667 | -11.86687 | 5.15354 | 0.54247 |
| D2 vs. D3 | -2.683 | -9.63446 | 4.26846 | 0.54247 |
| D2 vs. Stock | -4.89 | -13.89352 | 4.11352 | 0.54247 |
| D3 vs. Stock | -2.207 | -10.8717 | 6.4577 | 0.54247 |

Supplemental Table 1: Results from post hoc pairwise comparisons using a Benjamini-Hochberg correction comparing mean differences in plant height between inoculation treatments. Statistical significance( $p\leq0.05)$indicated with an asterisk.

| **Estimate** | **Estimated coefficients** | **Standard Error** | **Z value** | **P value** |
| --- | --- | --- | --- | --- |
| Negative control (Intercept) | 1.85 | 0.106 | 17.449 | 0 |
| D1 | -0.24 | 0.177 | -1.359 | 0.174 |
| D2 | -0.145 | 0.162 | -0.892 | 0.373 |
| D3 | -0.346 | 0.165 | -2.098 | 0.036* |
| Stock | -0.107 | 0.154 | -0.692 | 0.489 |

Supplemental Table 2: Coefficients for a log-linear (Poisson) regression of treatment level on leaf number. The estimated coefficients give us the estimated change in log odds compared to the reference level of the negative control. The z-value is the ratio of the estimated coefficient to its standard error. Statistical significance( $p\leq0.05)$indicated with an asterisk.
